# False rumours of disease outbreaks caused by infectious myonecrosis virus (IMNV) in the whiteleg shrimp in Asia

**DOI:** 10.1186/1477-5751-10-10

**Published:** 2011-08-03

**Authors:** Saengchan Senapin, Kornsunee Phiwsaiya, Warachin Gangnonngiw, Timothy W Flegel

**Affiliations:** 1 National Center for Genetic Engineering and Biotechnology (BIOTEC), National Science and Technology Development Agency (NSTDA), Pathumthani, 12120, Thailand; 2 Center of Excellence for Shrimp Molecular Biology and Biotechnology (Centex Shrimp), Faculty of Science, Mahidol University, Rama VI Rd., Bangkok, 10400, Thailand; 3 Department of Biotechnology, Faculty of Science, Mahidol University, Rama VI Rd., Bangkok, 10400, Thailand

## Abstract

**Background:**

Infectious myonecrosis virus (IMNV) disease outbreaks in cultivated whiteleg shrimp *Penaeus (Litopenaeus) vannamei *are characterized by gross signs of whitened abdominal muscles and by slow mortality reaching up to 70%. In 2006 the first disease outbreaks caused by IMNV in Asia occurred in Indonesia. Since then rumours have periodically circulated about IMNV disease outbreaks in other Asian countries. Our findings indicate that these are false rumours.

**Findings:**

Our continual testing by nested RT-PCR of shrimp samples suspected of IMNV infection from various Asian countries since 2006 has yielded negative results, except for samples from Indonesia. Our results are supported by the lack of official reports of IMNV outbreaks since January 2007 in the Quarterly Report on Aquatic Animal Diseases (QAAD) from the Network of Aquaculture Centers in Asia Pacific (NACA). In most cases, our shrimp samples for which tissue sections were possible showed signs of muscle cramp syndrome that also commonly causes muscle whitening in stressed whiteleg shrimp. Thus, we suspect that most of the false rumours in Asia about IMNV outside of Indonesia have resulted because of muscle cramp syndrome.

**Conclusions:**

Results from continual testing of suspected IMNV outbreaks in Asian countries other than Indonesia since 2006 and the lack of official country reports of IMNV outbreaks since January 2007, indicate that rumours of IMNV outbreaks in Asian countries outside of Indonesia are false. We suspect that confusion has arisen because muscle cramp syndrome causes similar signs of whitened tail muscles in whiteleg shrimp.

## Findings

### Origin of IMNV

Infectious myonecrosis virus (IMNV) is a double-stranded RNA (dsRNA) virus in the family *Totiviridae *near the genus *Giardiavirus*. Disease outbreaks in the whiteleg shrimp *Penaeus *(*Litopenaeus*) *vannamei *caused by this virus were first reported from Brazil in 2002 [1] and were characterized by gross signs of whitened abdominal muscles in the shrimp and by slow mortality persisting throughout culture (cumulative mortality reaching up to 70%). The causative virus was described in 2006 [2]. The viral particle is icosahedral and about 40 nm in diameter and the length of the whole genome is 7650 base pairs (GenBank AY570982). Although the black tiger shrimp (also called giant tiger shrimp) could be infected with IMNV in the laboratory, it did not die from the infection [3].

At the end of June 2006, Centex Shrimp received shrimp samples from a suspected IMNV outbreak in Indonesia. The samples tested positive for IMNV using the IQ2000 kit (GeneReach Corp, Taiwan) and our nested RT-PCR method [4]. Whole genome sequencing of the Indonesian samples revealed 99% identity to IMNV from Brazil. This strongly suggested that the source of the virus for the outbreak was living shrimp imported from Brazil, probably as broodstock for post-larval production. As previously reported [4], a contact in Indonesia who wished to remain anonymous related that *P. vannamei *broodstock had been smuggled onto Java island from Brazil for use in a commercial hatchery.

### Suspected IMNV disease outbreaks in Asia outside Indonesia

Since the report of IMNV outbreaks in Indonesia was published, false rumours have periodically circulated from China, Malaysia, Thailand and Vietnam claiming that IMNV outbreaks have also occurred there. The ultimate source of the false rumours is not known, but they may have resulted because other factors can cause muscle whitening in the whiteleg shrimp *P. vannamei *and lead to confusion, if subsequent tests are not carried out to determine the cause. In addition, the 6th edition of the Manual of Diagnostic Tests and Vaccines for Aquatic Animals incorrectly cited the publication above [4] as the authority for occurrence of IMNV outbreaks in Thailand. This error has now been corrected in the latest current on-line version of the manual.

**Figure 1:**
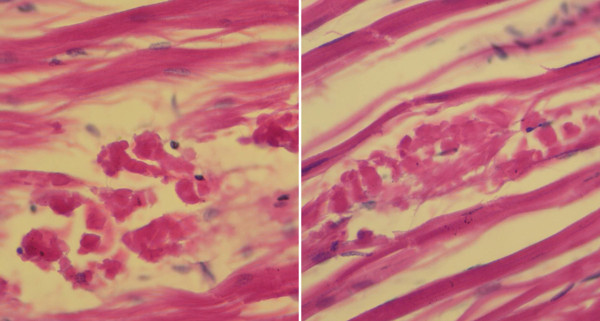
**Histology of muscle cramp syndrome**. Photomicrographs of shrimp muscle tissue showing clumping of muscle fibers (coagulative necrosis), similar to the clumping that occurs with IMNV infections but in the absence of accumulated shrimp blood cells and viral inclusions that are characteristic of IMNV lesions.

Prior to and especially after the IMNV outbreak in Indonesia, Centex Shrimp received many samples of shrimp with whitened muscles from shrimp cultivation ponds showing unusual mortality in China (including Taiwan), India, Malaysia, Thailand, Vietnam and Indonesia. We received the samples because the farm owners suspected that IMNV might have been the cause. All samples for RT-PCR testing consisted of pleopods collected from living shrimp and preserved in 95% ethanol. RNA was extracted and tested within 7 days of sample collection. This protocol was the same as that used for the original samples in which IMNV was detected from Indonesia. We have continually tested such samples since 2006 (Table 1) and as recently as June 2011 from Vietnam. All of these samples (except for samples from Indonesia) gave negative results for IMNV using both the IQ2000 detection system and our nested RT-PCR method [4]. The IQ2000 negative tests all showed an internal control band at 680 bp indicating that the RNA in each sample was intact, and the kit positive control lanes on the same gels gave the expected positive results. The negative results from Thailand have been confirmed by the Thailand Department of Fisheries (unpublished). In addition, at Centex Shrimp we have tested all of these samples since 2006 (as they arrived or as archived material) for *Penaeus vannamei *nodavirus (*Pv*NV), another virus reported to cause whitened muscles in whiteleg shrimp in the Americas [5], and all were also negative for *Pv*NV (Table 1). In most cases, the shrimp samples for which tissue sections were possible showed signs of muscle cramp syndrome [6] that also commonly causes whitened muscles in white shrimp under stressful situations. These are characterized by coagulative muscle necrosis (Figure 1) that also occurs with IMNV infections. However, the difference is that the coagulative necrosis in muscle cramp syndrome is not accompanied by the presence of hemocytic aggregation and cytoplasmic viral inclusions characteristic of IMNV infections [2]. Thus, we suspect that most of the false rumours in Asia about IMNV outside of Indonesia may have resulted because of muscle cramp syndrome.

**Table 1:** Source of samples and test results

		IMNV		*Pv*NV
**Country/Date** **(dd/mm/yr)**	**Total samples**	**Negative**	**Positive**	**Positive**

**Indonesia**				
26/06/06	4	0	**4**	0
04/10/06	15	0	**15**	0
28/11/06	2	2	0	0
04/05/07	10	4	**6**	0
08/06/07	8	4	**4**	0
10/06/09	20	20	0	0
26/06/09	5	0	**5**	0
09/07/09	3	2	**1**	0
20/10/09	7	0	**7**	0
29/03/10	2	2	0	0

**Thailand**				
15/09/06	7	7	0	0
02/10/06	24	24	0	0
14/03/07	4	4	0	0
03/07/09	6	6	0	0
04/08/10	2	2	0	0
05/08/10	2	2	0	0
11/08/10	6	6	0	0
17/09/10	3	3	0	0
03/05/11	8	8	0	0

**China**				
24/07/06	3	3	0	0
10/04/08	8	8	0	0
04/12/09	7	7	0	0
22/12/09	10	10	0	0
22/01/10	2	2	0	0
16/03/10	3	3	0	0

**Malaysia**				
22/08/06	5	5	0	0

**Taiwan**				
10/09/07	3	3	0	0

**Vietnam**				
07/11/06	2	2	0	0
25/01/10	3	3	0	0
21/06/11	4	4	0	0

**India**				
20/02/09	3	3	0	0
17/03/10	2	2	0	0

**Totals**	**193**	**151**	**42**	**0**

Source and test results for *P. vannamei *samples submitted by Asian shrimp farmers and tested for IMNV and *Pv*NV from the years 2006-2011.

### Possibility of false positive RT-PCR detection results

**Figure 2:**
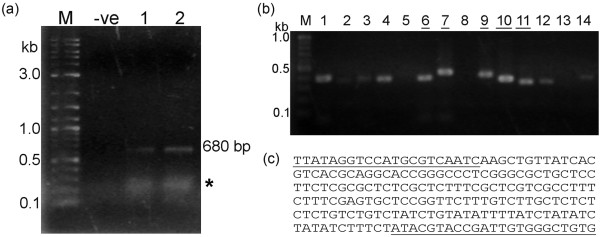
**Example of false positive RT-PCR results**. Example agarose gels of smeared amplicons from some shrimp samples. **(a) **Smeared amplicons (*) obtained from 2 shrimp samples tested for IMNV using the IQ2000 kit. The band at 680 bp is the kit internal control; -ve = negative control; M = DNA marker (2-log ladder, New England Biolabs). **(b) **Colony PCR screening of recombinant clones obtained from the bands similar to those  marked by an asterisk (*) in (a). Underlined numbers represent clones subjected to sequence analysis. **(**c**)** Sequencing result for clone 10 in (b) with a 204 bp-insert. Portions of the sequence with homology to IMNV are underlined.

While carrying out RT-PCR tests using the IQ2000 detection system, we noticed that RNA samples derived from shrimp pleopods (swimming legs) sometimes yielded weak, smeared bands around the expected size of the nested product (255 bp) of the kit (Figure 2a). When these products were purified from agarose gel and ligated into pDrive cloning kit (Qiagen) followed by colony PCR using vector primers (i.e., Sp6 and T7 promoter primers), variable insert sizes (ranging from 176-275 bp) were found among the tested recombinant clones (Figure 2b). Sequencing of 5 individual clones revealed that only short sequences (~21-23 bp) at the 3' and/or 5' ends of these inserts shared identity with IMNV (Figure 2c) and probably represented the sequences of primers used in the IQ2000 kit (sequences not revealed by the kit manufacturer). BLAST search results for the portions of these inserts excluding the putative IMNV kit primer sequences at each end revealed that 2 clones had no similarity to any record at GenBank while 3 clones matched sequences in the database. Of these 3 clones, 2 clones matched crustacean actin (27 out of 30 bp of GenBank number GU732815 and 24 out of 24 bp of FE087111) while the other matched a repeat sequence in the honey bee (GenBank accession number BI511369 nucleotides 11-184 with 50% coverage and 77% identity) and a similar sequence in a *P. monodon *shrimp EST library (GenBank accession number GW421137 nucleotides 217-315 with 67% coverage and 71% identity). Figure 2c depicts the sequence of the latter clone. These smeared bands were not obtained using our RT-PCR method [4] or with either method when using RNA extracts from internal organs of the same shrimp that gave smeared bands using pleopod extracts. Because of this experience, we believe that the spurious bands may have arisen from contaminating DNA arising from epifauna or debris attached externally to the shrimp pleopods. To avoid this problem, we recommend that pleopods be avoided and that internal samples such as hemocytes or muscle tissue (the location of IMNV lesions) be used instead to prepare RNA extracts for IMNV detection by RT-PCR assay. This would avoid DNA contamination from epifauna sometimes attached to the shrimp cuticle.

### Additional support for falseness of rumours

Since the total number of specimens we have received and tested from Thailand and China is relatively small, and from India, Malaysia and Vietnam is very small, it might be suggested that our sampling was insufficient to claim absence of IMNV from these countries. However, it must be kept in mind that these were not random samples but samples selected by farmers and technical consultants because they came from events outside the normal shrimp cultivation experience in their respective countries, including experience with other diseases. In addition, since January 2007, the Quarterly Report on Aquatic Animal Diseases (QAAD) from the Network of Aquaculture Centers in Asia Pacific (NACA) has included infectious myonecrosis caused by IMNV in its list of reportable diseases from competent authorities for aquatic animal diseases in 18 member countries in Asia. Its members include China, India, Malaysia, Thailand, Vietnam and Indonesia. Unfortunately, the numbers of specimens tested and found negative by these authorities is not given. However, the presence of QAAD reports on the occurrence of IMNV from Indonesia but not from China, India, Malaysia, Thailand and Vietnam supports our contention that outbreaks of infectious myonecrosis have not yet occurred in Asia outside of Indonesia. Since NACA has a disease monitoring and reporting program in place and since its QAAD reports are freely accessible at http://www.enaca.org, we recommend that anyone wishing to check the validity of rumours of IMNV outbreaks (or disease outbreaks of other aquaculture species) refer to those reports.

### Recommendation to the shrimp industry

Without the import of infected, living shrimp for aquaculture, it is extremely unlikely that IMNV could come to Thailand or any other Asian country where it does not currently occur [7]. By contrast, it is our opinion that frozen, packaged shrimp from normal harvests destined for human consumption does not pose a threat, and that there would be no need to block its import, so long as appropriate measures are adopted to reduce the risk of diversion for unintended uses [8]. With respect to the reprocessing of bulk frozen whiteleg shrimp imported from Indonesia or Brazil, the situation is more complicated. It should be safe, so long as proper care is taken in disposing of the processing wastes. This must be overseen by the appropriate government agencies.

We would like to take this opportunity to warn everyone in the shrimp industry that import into any Asian country of shrimp broodstock and fry for aquaculture directly or indirectly from Brazil and Indonesia currently entails an extremely high risk of importing IMNV. Given the threat of extreme economic loss to shrimp farmers, it would be very self-serving and socially reprehensible for anyone to engage in such activities without undertaking the strictest quarantine measures. We hope that everyone in the Asian shrimp industry will refrain from doing this and will quickly inform their national competent authorities if they know of anyone attempting to do so.

## Conclusions

Negative test results for IMNV in our continual tests since 2006 and lack of official reports of IMNV outbreaks in the Asian member countries of NACA other than Indonesia since 2007, indicate that rumours of IMNV outbreaks in those countries are false. We suggest that most of the false rumours have resulted from mistaken diagnosis based on gross signs of whitened muscles probably caused by muscle cramp syndrome.

## Competing interests

The authors declare that they have no competing interests.

## Authors' contributions

SS and KP did all of the RT-PCR testing and WG prepared tissue sections for light microscopy. WG and TWF were responsible the hisopathological analysis. SS and TWF conceived the work and prepared the manuscript. All authors have read and approved the final manuscript.
